# Effects of gastrodin on the expression of brain aging‐related genes in SAM/P‐8 mice based on network pharmacology

**DOI:** 10.1002/ibra.12076

**Published:** 2022-11-06

**Authors:** Nan Zhao, Rui Jiang, Jun‐Jie Cheng, Qiu‐Xia Xiao

**Affiliations:** ^1^ Department of Anesthesia, Hospital of Stomatology Zunyi Medical University Zunyi China; ^2^ Department of Anesthesia Affiliated Hospital of Zunyi Medical University Zunyi China; ^3^ Department of Anesthesiology Nanchong Central Hospital Sichuan China; ^4^ Department of Anesthesiology University of Virginia Charlottesville Virginia USA

**Keywords:** aging, gastrodin, gene, molecular mechanism, network pharmacology

## Abstract

**Background:**

Gastrodin can reduce neuronal damage through multiple targets and pathways, and can be useful in preventing and treating degenerative lesions of the central nervous system, but the specific mechanism has not been elucidated.

**Methods:**

The aging‐related genes in the hippocampus and the frontal cortex were detected in adult and aged mice treated with gastrodin or not. In addition, we collected the target genes of gastrodin and aging from a network database, and a Venn diagram was created to obtain the intersection target genes of gastrodin and aging. Then, the String database was used to analyze the protein–protein interactions (PPIs) between aging‐related genes and the target genes of gastrodin and aging. The “drug–disease–target–pathway” network was constructed using Cytoscape 3.7.2 software, and the main mechanism and pathway of key genes were analyzed by Kyoto Encyclopedia of Genes and Genomes (KEGG) and Gene Ontology (GO). Finally, the reliability of these key genes was further verified by molecular docking technology.

**Results:**

The results showed that 6 out of 10 genes related to brain aging were differentially expressed after gastrodin intervention. Moreover, there were 11 key genes between gastrodin and differentially expressed genes related to brain aging. GO and KEGG results suggested that material metabolism and carbohydrate digestion and absorption were associated with the pathological mechanism of gastrodin antiaging. Molecular docking results also confirmed the good binding activity of gastrodin to the key genes.

**Conclusion:**

Gastrodin plays a potential role in antiaging by regulating substance metabolism and carbohydrate digestion and absorption.

## Graphical abstract

In this study, we first explored whether gastrodin affected the expression of aging‐related genes through animal experiments. Multiple databases were used to screen potential drug targets of gastrodin and disease targets of aging. A protein–protein interaction network is constructed to analyze the connection among these targets and identify the hub genes on the basis of the topological interaction between these targets. Afterward, an enrichment analysis of the Kyoto Encyclopedia of Genes and Genomes and Gene Ontology was performed to elucidate the biological functions of hub genes. Finally, molecular docking techniques were applied to confirm the reliability of these crucial targets.

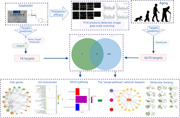

## 1 INTRODUCTION

Aging refers to an irreversible phenomenon wherein, when organisms mature, with age, their body' function, ability to stabilize the internal environment, and ability to stress declines; this is also accompanied by degenerative changes in multiple organs and tissue structures, leading to death.^1^ Current studies have shown that brain aging plays a dominant role in the aging process of the body, and the lesion sites are mainly concentrated in the hippocampus and the frontal cortex.^2^, ^3^ Among these, the hippocampus is one of the most vulnerable brain regions. Diseases such as Alzheimer's disease (AD), which are closely related to learning and memory, are directly related to the functional decline of the hippocampus and are accompanied by changes in specific gene expression.^4^, ^5^ With the aging of populations worldwide and the increasing incidence of aging‐related diseases, the social and economic burden is also increasing.^6^ At present, antiaging treatment mainly includes drug therapy, stem cell and microbial transplantation, hormone replacement therapy, and so forth. Unfortunately, the current treatment for AD has not shown significant clinical efficacy.^7^ In recent years, new antiaging strategies have led to new directions and hopes for treatment, but many difficulties still need to be overcome to achieve clinical application and treatment. Therefore, active exploration of the pathogenesis of aging, development of effective drugs to delay aging, and reduction of the incidence of aging‐related diseases are of great significance to improve people's living standards and quality of life.

Aging has remained an unresolved issue in the medical field. It is a multifactorial process that leads to a gradual decline in the function of cells, tissues, and organisms. Although aging cannot be stopped, it is possible to slow down the rate of aging through various methods. Traditional Chinese medicine (TCM) is receiving increasing attention in the prevention and treatment of various neurodegenerative diseases (NDs) caused by aging.^8^, ^9^, ^10^, ^11^, ^12^ Studies have shown that TCM can improve the neurocholine function of the body, protect cerebral vessels, and significantly improve the cognitive function of patients by acting on multiple tissues and multiple targets.^13^, ^14^ Gastrodin is a phenolic glycoside isolated from the rhizome of *Gastrodia elata* and its tuber has been used in TCM for centuries. Its active ingredients have physiological and health‐promoting features, including antitumor, memory improvement, and neuroprotective activities.^15^, ^16^ In particular, TCM is widely used in Asia to treat epilepsy, headache, amnesia, stroke, and other diseases.^17^, ^18^, ^19^ Recent studies have found that gastrodin can alleviate neuronal damage due to various causes by inhibiting intracellular calcium overload, inhibiting neuronal apoptosis, stabilizing neuron membrane, and antioxidation. It also plays potential roles in the prevention and treatment of NDs, including AD, Parkinson's disease, and vascular dementia.^20^, ^21^, ^22^, ^23^ Although gastrodin has shown great potential in the treatment of NDs, there are no in‐depth and specific studies on whether gastrodin can improve aging by regulating antiaging genes.

With the development of interdisciplinary fields such as bioinformatics, system biology, and comprehensive pharmacology, the research strategy for exploring the interaction between drugs and diseases has gradually shifted from independent research to systematic overall analysis.^24^ Network pharmacology based on the concept of “drug–disease–target–pathway” measures the regulation of drugs on biological molecular networks from a systematic and holistic perspective. Its basic principle coincides with the characteristics of multiple targets and multiple functions in the treatment of diseases by TCM.^25^ TCM network pharmacology, which aims to understand the network‐based biological basis of complex diseases, TCM syndromes, and herb treatments, plays a critical role in the origin and development process of network pharmacology.^26^ Therefore, the application of network pharmacology based on systematic bioinformatics has unique advantages in studying the molecular mechanism of TCM compounds.^27^

**Figure 1:**
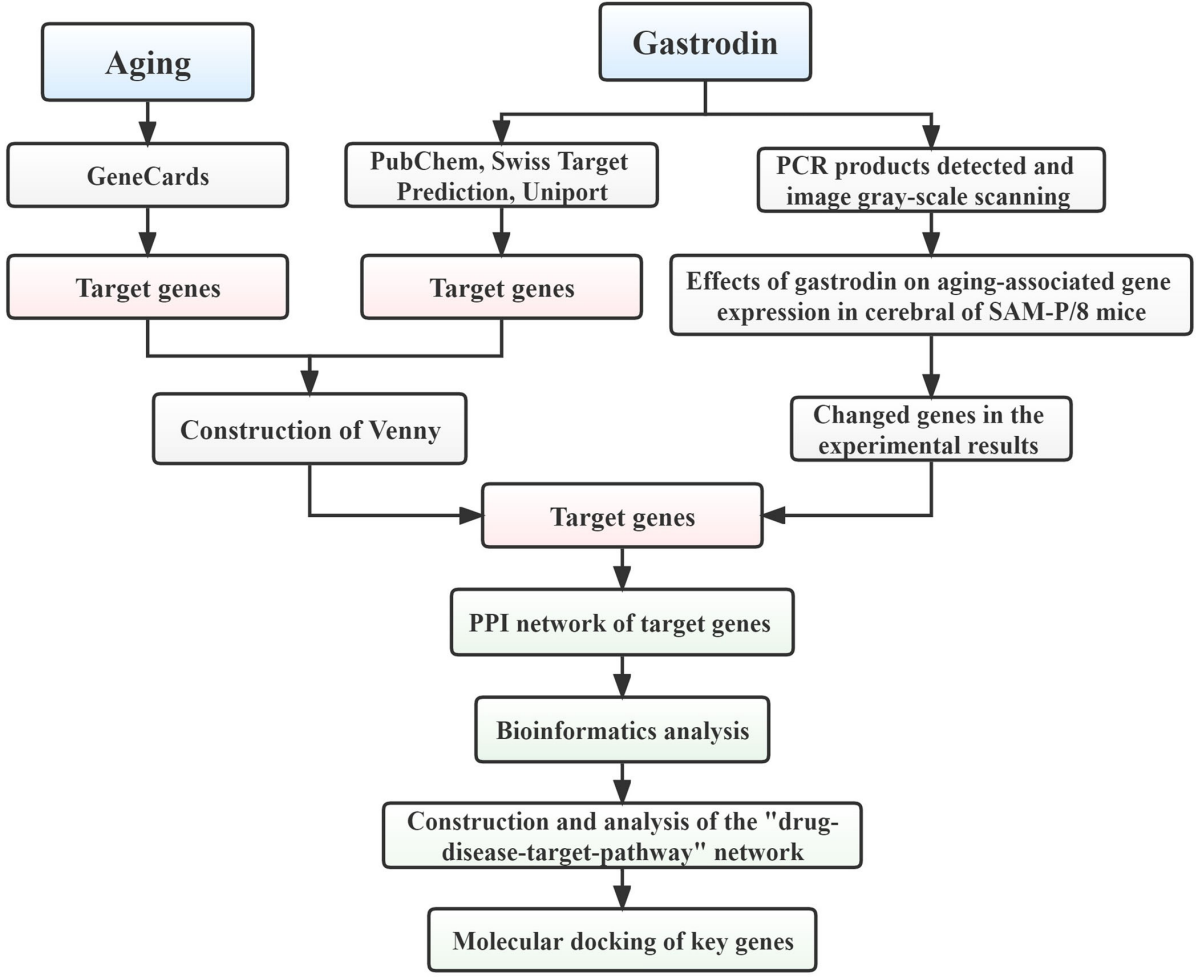
Flowchart of the study design [Color figure can be viewed at wileyonlinelibrary.com]

Consequently, the use of new analysis methods to explore the key genes and mechanisms of antiaging of TCM will provide a new avenue for subsequent research and application of TCM. In this study, we first explored whether gastrodin affected the expression of aging‐related genes through animal experiments and then further explored the antiaging targets and possible mechanisms of gastrodin in combination with network pharmacology. At the same time, we also constructed a potential “drug–disease–target–pathway” network of gastrodin and identified the key genes by molecular docking technology to provide a theoretical and experimental basis for subsequent research (Figure 1).

## 2 MATERIALS AND METHODS

### 2.1 Animal grouping and treatment

The animal experimental design in this study was approved by the Animal Experimental Ethics Review Committee of Kunming Medical University (approval no. kmmu20221547). SAM‐P/8 mice were purchased from the Experimental Animal Center of Tianjin University of TCM. The adult mice were 7 months old and the elderly mice were 13 months old, with a weight of (25 ± 2) g. The mice were kept in a room with the temperature maintained at 22 ± 2.0°C and humidity maintained at 50 ± 5%, with light exposure for about 12 h a day and free access to food and water. Twenty SAM‐P/8 mice were randomly divided into four groups (*n* = 5/group): adult control group, adult gastrodin group, aged control group, and aged gastrodin group. The gastrodin injection (H20013046; Kunming Pharmaceutical Group) was diluted with medical 0.9% normal saline at 5 mg/ml, and mice in the adult gastrodin group and the aged gastrodin group were administered an intraperitoneal injection at a dose of 1 g/kg, once a day, for 14 consecutive days, with the left and right abdominal cavity alternated every other day. Neither the adult control group nor the aged control group received any treatment.

### 2.2 Tissue harvest

After 14 days of feeding, mice in each group were intraperitoneally injected with 1% pentobarbital sodium 30 mg/kg. The disappearance of the tail clamp reflex indicated that anesthesia was effective. Perfusion was performed with precooled saline and a 4% paraformaldehyde phosphate buffer solution (pH = 7.2) after left ventricular catheterization. The mice were intraperitoneally injected with 1% pentobarbital sodium at 50 mg/kg. After the tail pinch reflex disappeared, the anesthesia took effect, and then the perfusion was performed. Hippocampus and frontal cortex tissues were rapidly separated and rinsed with precooled diethyl pyrocarbonate‐treated water, placed into a 1.5 ml Eppendorf tube containing 1 ml of TRIzol lysis RNAase‐free, and homogenized in an ice bath.

### 2.3 Reverse transcription‐polymerase chain reaction (RT‐PCR)

RNA was isolated according to the instructions of the kit. The concentration and purity of RNA were detected by agarose gel electrophoresis and an ND‐1000 spectrophotometer (UV‐250IPC Shimadzu). Total RNA was isolated by TRIzol reagent (Takara Bio Inc.) and complementary DNA (cDNA) was synthesized according to the RevertAidTM First Strand cDNA Synthesis Kit (Thermo Scientific). The first strand of synthesized cDNA can be directly used for PCR amplification. According to the instructions of the 2× PCR Master Mix Kit (#K0171; Fermentas), *β*‐actin and 10 aging‐related genes were amplified, and the primer sequences are shown in Table 1. The total volume of each reaction was 20 μl: 10 μl of 2× PCR Master Mix, 8 μl of PCR water nuclease‐free, 0.5 μl of upstream primer, 0.5 μl of downstream primer, 1 μl of RT cDNA template. Thermal cycle parameters were as follows: 94°C denaturation for 5 min, 94°C denaturation for 1 min, annealing for 1 min, 72°C extension for 1 min, and 35 cycles after 72°C extension for 10 min. PCR products were detected by 1% agarose gel electrophoresis, and gel images were taken under the ultraviolet mode of the BIO‐GEL gel imager to determine the gray value of each target gene band (WD‐9403c; Beijing Liuyi Instrument Factory). Quantity One software was used to analyze electrophoresis bands and calculate the ratio of target gene bands to the *β*‐actin gray value.

**Table 1:** Gene primer sequence, renaturation temperature, and product length

Gene	Primer sequence	Renaturation temperature (°C)	Product length
SCN2B	Sense	56	355
	5′‐CTACACCGTGAACCACAAGCA‐3′		
	Antisense		
	5′‐GACCACAGCCAGGAAACCC‐3′		
MAP2	Sense	53	427
	5′‐AGGAAGCAGCAAGTGGTGAC‐3′		
	Antisense		
	5′‐TTTGGAGGAGTGCGGATG‐3′		
Sortilin‐1	Sense	52	376
	5′‐CGCTACCGCAAAGAACAA‐3′		
	Antisense		
	5′‐GGAAGCAAGCCCAGTGAA‐3′		
Rab6A	Sense	52	465
	5′‐GTCCTTGATCACCCGATTC‐3′		
	Antisense		
	5′‐TCCTGTGTGCTTTCCATTC‐3′		
Calcineurin‐B	Sense	52	479
	5′‐ATGGGAAATGAGGCGAGTT‐3′		
	Antisense		
	5′‐AGGCCACCTACGACAGCAC‐3′		
MAPKK4	Sense	51	298
	5′‐ATTGCCCATACATTGTTCAGT‐3′		
	Antisense		
	5′‐ATCAGAGCGGACATCATACC‐3′		
MAP1B	Sense	53	146
	5′‐TGCCCGCCATAAACTGC‐3′		
	Antisense		
	5′‐GGTGGGTGGTGCTTAGGAG‐3′		
Regucalcin	Sense	52	378
	5′‐AGTTGGGAGGCTATGTTGC‐3′		
	Antisense		
	5′‐TGCGGTTGGAAATCTGTC‐3′		
RAP2A	Sense	49	159
	5′‐CCTGGTCGGGAACAAAGT‐3′		
	Antisense		
	5′‐TTCATCTGCCGCACAATT‐3’		
*β*‐actin	Sense	58	517
	5′‐ATATCGCTGCGCTGGTCGTC‐3’		
	Antisense		
	5′‐AGGATGGCGTGAGGGAGAGC‐3′		

### 2.4 Network pharmacology analysis

#### 2.4.1 Screening of gastrodin and aging targets

The chemical structure of gastrodin was obtained from the chemical information database PubChem using “Gastrodin” as the keyword and its “Canonical SMILES” sequence was found (https://pubchem.ncbi.nlm.nih.gov/). Swiss Target Prediction (http://swisstargetprediction.ch/) is a website that can predict possible targets according to the chemical structure of drug components. The canonical SMILES sequence of gastrodin was imported into the website for target prediction. In addition, the collected targets were converted into target names and gene names in the Uniport database (https://www.uniprot.org/). With “aging” as the keyword, all relevant disease targets were found in the GeneCards database (https://www.genecards.org/) and downloaded for analysis. After taking two medians of correlation, targets with correlations >1.43 were identified.

#### 2.4.2 Core target analysis and protein–protein interaction (PPI) network construction

The screened gastrodin and aging‐related targets were uploaded to the plotting website Jvenn (http://www.bioinformatics.com.cn/static/others/jvenn/example.html), and the Venny diagram was plotted to find the common targets of drugs and diseases. These common targets were the required core targets. String (https://string-db.org/) is a website that can analyze the relationship between proteins online. The String database was used to input 11 cross‐linked targets of gastrodin and aging together with six changed targets in the experimental results into the String to search and continue to produce protein interaction maps. The high‐definition interaction diagram and the interaction table were exported and downloaded. The interaction table was imported into Cytoscape 3.7.2. According to the degree value and combined_score, nodes with different sizes and connection lines with different thicknesses were generated to draw a PPI network diagram.

#### 2.4.3 Gene ontology (GO) functional enrichment analysis and Kyoto Encyclopedia of Genes and Genomes (KEGG) pathway analysis

In this study, the common targets of gastrodin and aging were analyzed by bioconcentration, and the genes and gene products were annotated by GO analysis according to the biological process (BP), the cellular component (CC), and the molecular function (MF). KEGG is a useful resource for the systematic analysis of gene function and related high‐level genomic function information. Therefore, to further clarify the function of the selected intersection genes and their roles in the signal transduction pathway, GO function analysis and KEGG pathway enrichment analysis were performed using the DAVID database (http://david.abcc.ncifcrf.gov/), and the *p* value and fold enrichment value were determined.

#### 2.4.4 Molecular docking

Molecular docking has become an important technology for computer‐aided drug research in recent years. It can be used to evaluate the interaction between active ingredients and core targets and predict their binding modes and affinity. The three‐dimensional structure file of the core protein was obtained from the RCSB protein database (http://www.rcsb.org/). The crystal structure of the protein was initialized using AutoDockTools software (version: 1.5.6), including removal of solvent molecules and ligands, addition of polar hydrogen, energy initialization, and so forth, and converted into the pdbqt format file required for docking. The three‐dimensional structure of gastrodin was optimized by the MM2 force field using Chem3D Pro 14.0 software (version: 14.0.0.117), and the number of rotatable keys was set by AutoDockTools software and converted into the pdbqt format file required for docking. Molecular docking scoring was performed using the molecular docking software AutoDock Vina (version: 1.1.2).

### 2.5 Statistical analysis

All data in this study were processed using SPSS 21.0 statistical software, and all measurement data were expressed as mean ± standard deviation ($\bar{x}$ ± SD). The ratio of the average optical density of each gene *β*‐actin between the two groups was tested using an independent‐sample *t*‐test, and *p* < 0.05 indicated that the difference was statistically significant.

## 3 RESULTS

### 3.1 Effects of gastrodin on aging‐related genes

**Figure 2:**
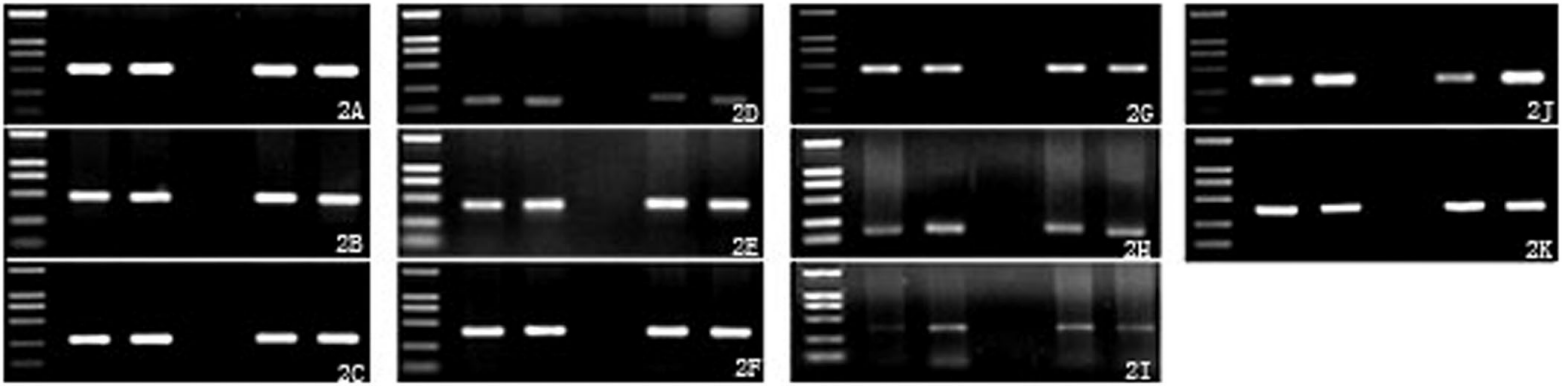
One percent agarose gel electrophoresis of polymerase chain reaction products of *β*‐actin and aging‐related genes in the hippocampus. (A) *β*‐Actin, (B) Calcineurin‐B, (C) Calmodulin‐1, (D) MAP1B, (E) MAP2, (F) MAP2K4, (G) Rab6A, (H) RAP2A, (I) Regucalcin, (J) SCN2B, (K) Sortilin‐1. The swimming lanes from left to right are the DL2000 marker (2000, 1000, 750, 500, 250, and 100), the adult control group, the adult gastrodin group, the aged control group, and the aged gastrodin group.

**Figure 3:**
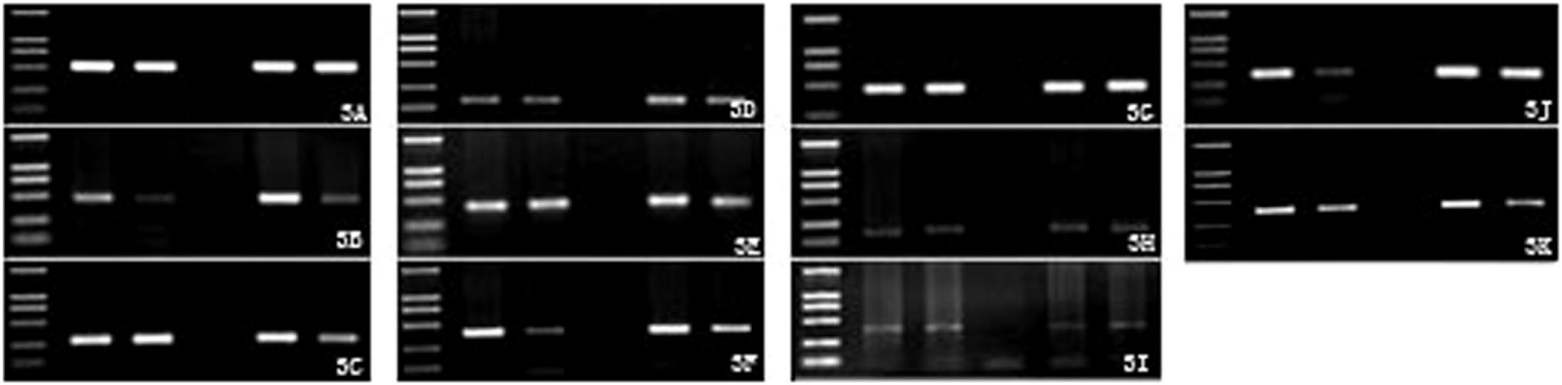
One percent agarose gel electrophoresis of polymerase chain reaction products of *β*‐actin and aging‐related genes in the frontal cortex. (A) *β*‐Actin, (B) Calcineurin‐B, (C) Calmodulin‐1, (D) MAP1B, (E) MAP2, (F) MAP2K4, (G) Rab6A, (H) RAP2A, (I) Regucalcin, (J) SCN2B, (K) Sortilin‐1. The swimming lanes from left to right are the DL2000 marker (2000, 1000, 750, 500, 250, and 100), the adult control group, the adult gastrodin group, the aged control group, and the aged gastrodin group.

**Figure 4:**
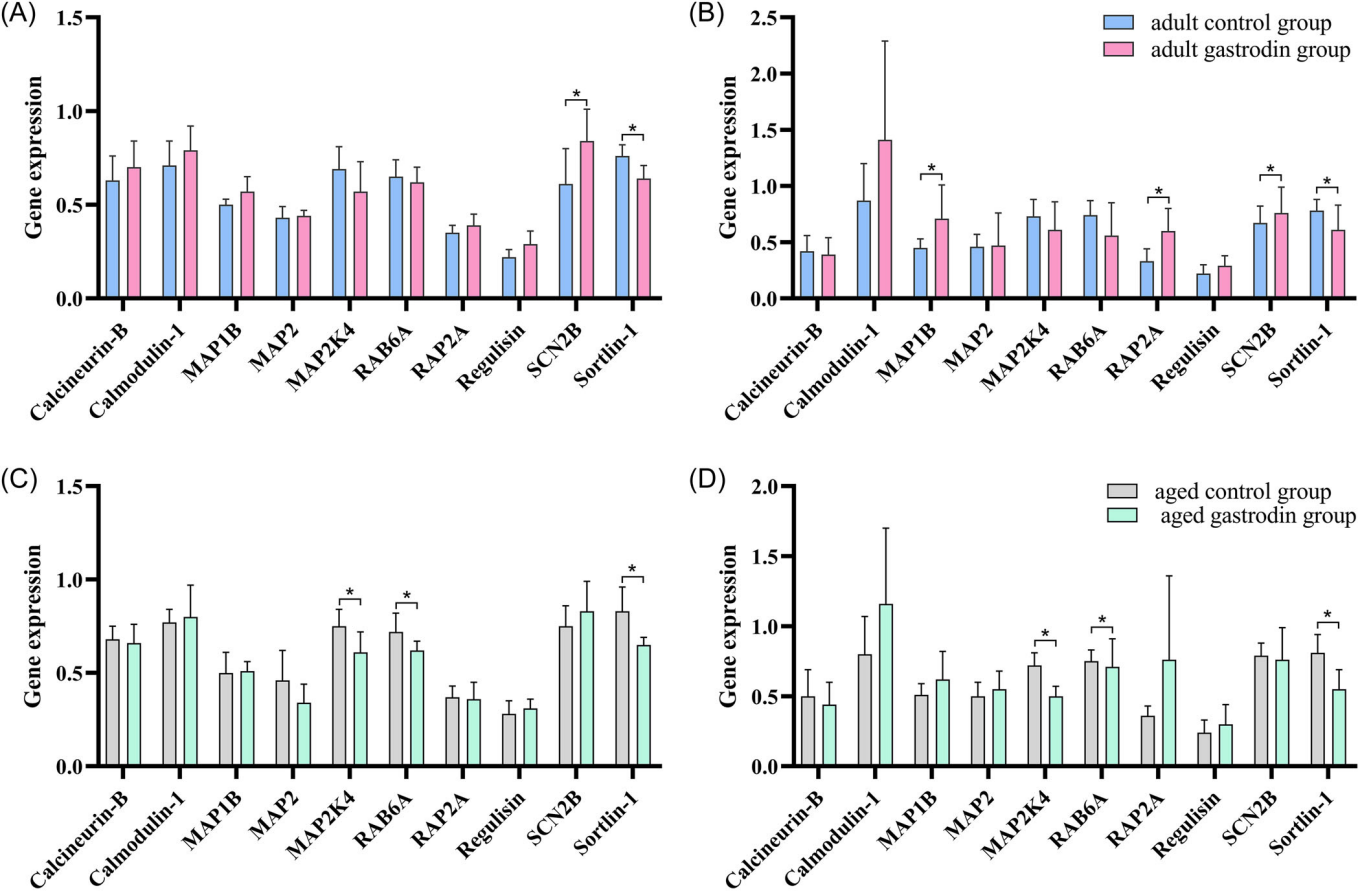
Expression of aging‐related genes in SAM‐P/8 mice. (A) Gene expression in the hippocampus of the adult control group and the gastrodin group. (B) Gene expression in the frontal cortex of the adult control group and the gastrodin group. (C) Gene expression in the hippocampus of the aged control group and the gastrodin group. (D) Gene expression in the frontal cortex of the aged control group and the gastrodin group. *p < 0.05. [Color figure can be viewed at wileyonlinelibrary.com]

The expression of β‐actin and aging‐related genes was detected in the hippocampus and the frontal cortex of all groups, and the target gene fragment was obtained, which was consistent with the expected fragment length (Figures 2 and 3). Gray‐scale scanning was performed on the image, and the ratio of the image to *β*‐actin was calculated after data correction for statistical analysis. The results showed that these 10 genes were expressed in the hippocampus and the frontal cortex of SAM‐P/8 mice treated with gastrodin, although the levels were different. These 10 genes may be involved in maintaining the physiological functions of the hippocampus and the frontal cortex, and the maintenance of their expression may be necessary in the aging process of the hippocampus and the frontal cortex in mice, but the specific functions are different. We compared the adult gastrodin group with the adult control group and the aged gastrodin group with the aged control group. The results are shown in Figure 4. Compared with the control group, the expression of SCN2B was increased and the expression of Sortilin‐1 was decreased in the hippocampus and the frontal cortex of adult mice treated with gastrodin. The expression of MAP1B and RAP2A increased in the frontal cortex (Figure 4, *p* < 0.05). Compared with the control group, the expression of MAP2K4, Sortilin‐1, and Rab6A in the hippocampus and the frontal cortex of aged mice decreased (Figure 4, *p* < 0.05).

### 3.2 Screening of key targets and analysis of biological functions

#### 3.2.1 Acquisition of the intersectional core target genes of gastrodin and aging

**Figure 5:**
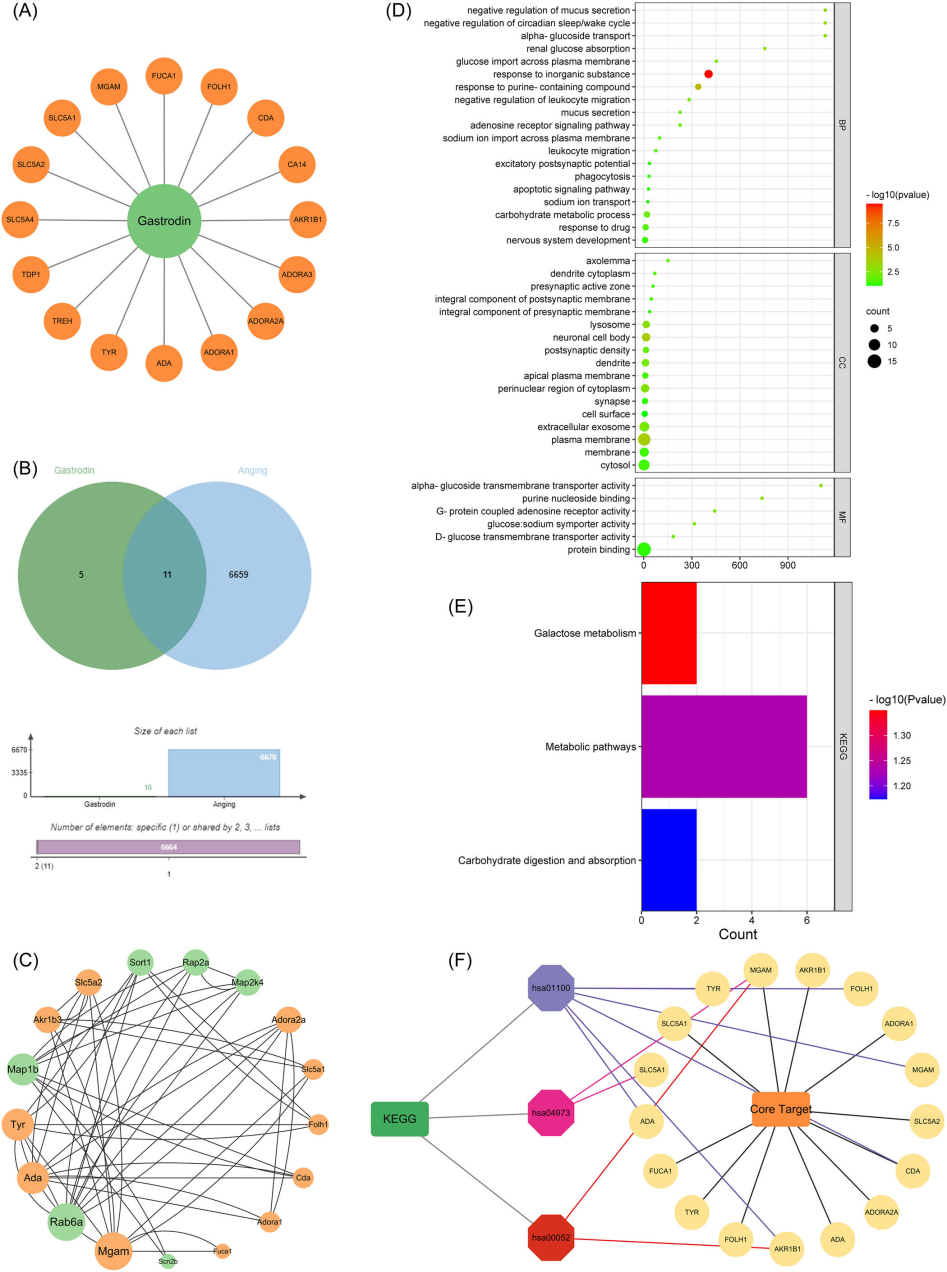
Screening of key targets and biological function analysis. (A) Core targets of gastrodin. (B) Wayne plot of the intersection target of gastrodin and aging. Green and blue circles represent the screened gastrodin and aging targets, respectively, and the middle part represents the common target of gastrodin and aging. (C) Interaction map of intersection targets and experimental targets. The larger the node area, the more important it is in the network. (D) Gene Ontology enrichment analysis of key genes of gastrodin antiaging. (E) Enrichment analysis of the Kyoto Encyclopedia of Genes and Genomes pathway of gastrodin antiaging key genes. (F) “Target–pathway” network diagram of gastrodin antiaging. [Color figure can be viewed at wileyonlinelibrary.com]

Through database retrieval, 16 target genes of gastrodin were screened (Figure 5A). There were more than 20,000 target genes related to aging. After taking two medians of correlation, 6670 target genes with a correlation >1.43 were determined. The Venny map was created using an online drawing website, and 11 core target genes of gastrodin and aging were obtained, including TYR, CDA, ADORA2A, SLC5A2, ADORA1, AKR1B3, SLC5A1, FUCA1, MGAM, FOLH1, and ADA (Figure 5B).

#### 3.2.2 PPI network of target genes

The PPI network can be used to discover multiple interactions among multiple proteins in diseases and determine potential therapeutic targets.^28^ The interaction between 11 selected genes and 6 changed genes in the experimental results was determined using the String database to determine the mode of interaction and the score between nodes, and the PPI network was constructed. Cytoscape 3.7.2 was used to set up the connection lines with different sizes and different thicknesses according to the degree value and combined_score, and the PPI network diagram was constructed. Green represents the gene mentioned in the experimental results and orange represents the gene of the core target. Furthermore, the size of each point was proportional to its participation in the network (Figure 5C). The results showed that the main core genes in the network included Mgam, Rab6a, and Ada, which were mainly involved in starch digestion and hydrolysis, protein transport regulation, and purine metabolism.

#### 3.2.3 Bioinformatics analysis

To explore the biological function of core targets, GO function and KEGG pathway enrichment analyses were performed. Using the DAVID database, GO enrichment analysis of 17 target genes involved in the PPI network was carried out, and 42 enrichment results were obtained, including 19 BP items, 17 CC items, and 6 MF items (Figure 5D). Similarly, the KEGG pathway analysis of key gene genes showed that the antiaging mechanism of gastrodin was mainly related to the three pathways of galactose metabolism, metabolic pathways, and carbohydrate digestion and absorption (Figure 5E). The 17 key targets and pathways of gastrodin were mapped to the “target–pathway” network by Cytoscape 3.7.2 software. To establish the network more conveniently, the pathway of gastrodin is represented by hsaID. Based on the network analysis, the network topology characteristics of the network model are analyzed (Figure 5F). The results showed that the core pathways, including galactose metabolism, metabolic pathways, and carbohydrate digestion and absorption, were the potential therapeutic processes of gastrodin in delaying aging. Seventeen genes are interrelated, which may be the key target genes for gastrodin to delay aging.

### 3.3 Molecular docking of key genes

**Figure 6:**
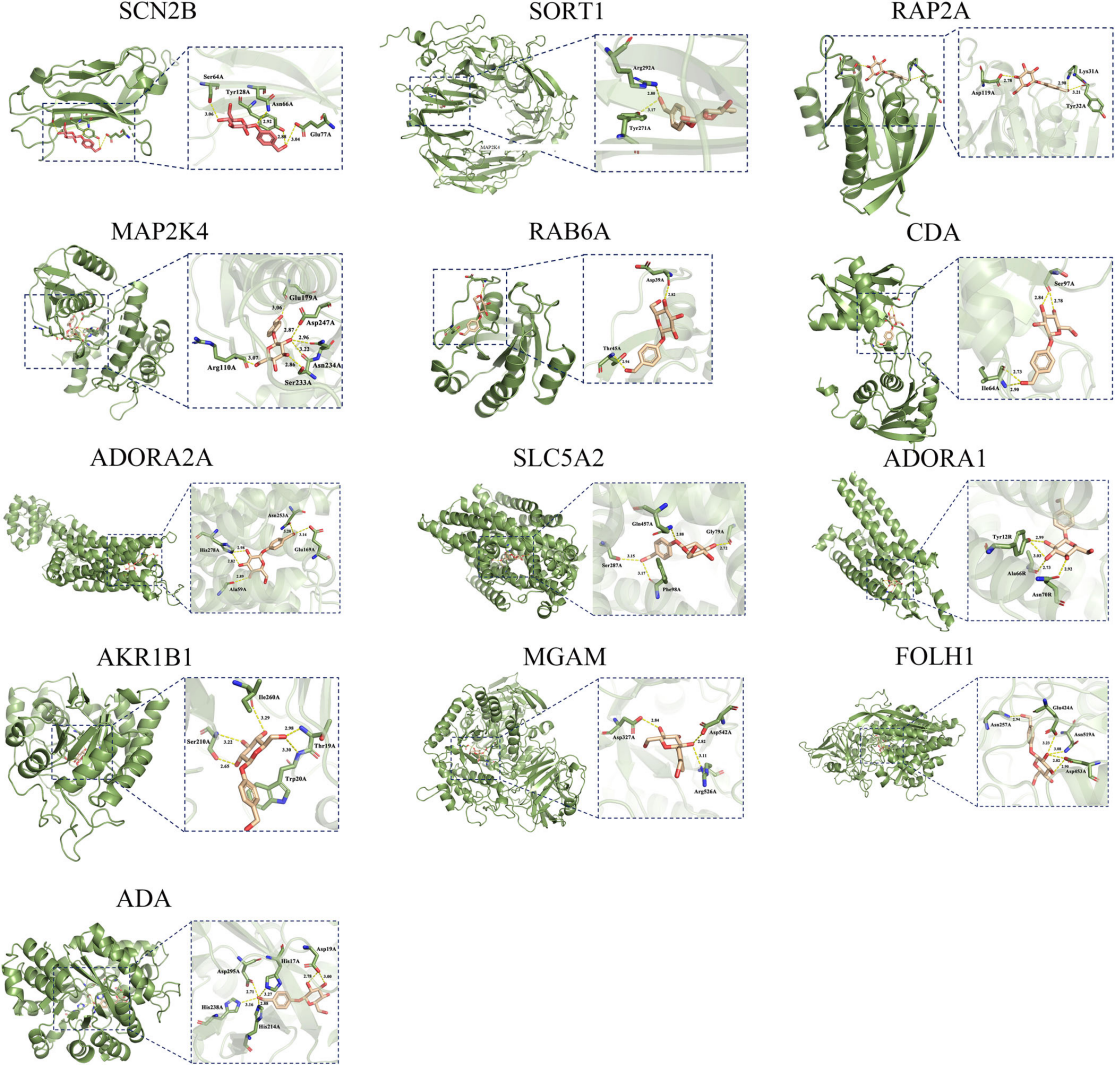
Molecular docking diagram of gastrodin and key targets [Color figure can be viewed at wileyonlinelibrary.com]

In this study, the possible interactions between 17 key target proteins and gastrodin in the experimental and network pharmacology analysis results were verified by molecular docking. AutoDock Vina software was used to perform molecular docking between gastrodin and 17 proteins such as SCN2B (Figure 6). The docking results were expressed as the affinity score. The smaller the value, the stronger the binding force. When affinity ≤ −7 kcal/mol, it showed strong binding activity.^14^ In this experiment, MAP1B, TYR, SLC5A2, and ADORA1 had no protein structure docking. Among the 13 docking proteins, the docking scores were less than −7 kcal/mol, except for SORT1, MGAM, and ADA proteins, indicating that gastrodin may have good binding activity with these proteins. The above results suggest that the expression of these proteins may play an important role in the antiaging of gastrodin (Table 2).

**Table 2:** Molecular docking information of key targets

Proteins	PDB ID	Affinity (kcal/mol)	Center_*x*	Center_*y*	Center_*z*
SCN2B	6VRR	−7.3	−16.7	−0.3	1.8
SORT1	5MRH	−6.6	−31.6	40.1	13.4
MAP1B	‐	‐	‐	‐	‐
RAP2A	1KAO	−7	−9.2	0.8	5
MAP2K4	3ALO	−7	−22.9	−12.7	11
RAB6A	1YZQ	−7.7	24.8	17.4	14.2
TYR	‐	‐	‐	‐	‐
CDA	1MQ0	−7.6	31.7	93.3	106.1
ADORA2A	3EML	−7.6	−9.1	−7.1	55.9
SLC5A2	7VSI	−7.9	38.3	50.2	46.4
ADORA1	7LD4	−7.2	87.5	71.3	79.8
AKR1B3	1IEI	−8.1	−5.1	0.2	9.9
SLC5A1	‐	‐	‐	‐	‐
FUCA1	‐	‐	‐	‐	‐
MGAM	3CTT	−6.6	1.3	−15.7	20.2
FOLH1	2JBJ	−8.5	16.3	45.3	42
ADA	3IAR	−6.9	6.7	−3.3	0.1

## 4 DISCUSSION

In this paper, the molecular mechanism of gastrodin antiaging was explored by combining experimental verification using mice and network pharmacology analysis. The experimental results obtained from mice showed that gastrodin could regulate brain aging‐related genes, which might act on target proteins SCN2B, Sortilin‐1, MAP1B, RAP2A, MAP2K4, and Rab6A. To further explore the molecular mechanism by which gastrodin improves aging, the potential targets and mechanism of gastrodin antiaging were analyzed by screening, target prediction, and bioinformatics analysis using network pharmacology and a public database. From the results of network pharmacology analysis, 11 gastrodin and aging intersection genes were constructed with six genes changed in the experiment to construct PPI, and GO and KEGG analyses were performed to explore the three most relevant pathways. The “drug–disease–target–pathway” diagram was drawn and verified by molecular docking technology. The results showed that 10 of the 17 proteins had high binding activity to gastrodin.

SCN2B can affect the formation and transmission of neuronal action potential by regulating the sodium ion channels, which plays an important role in the maintenance of neuronal physiological functions, and is related to aging and memory decline.^29^, ^30^ Sortilin‐1, a transmembrane neuropeptide receptor encoded by the SORT1 gene, is a key molecule regulating neuronal activity and function.^31^ It not only contributes to the functional integrity of the nervous system under physiological conditions but also plays an important role in neuronal injury and disease.^32^ MAP1B is the first microtubule‐associated protein expressed during the development of the nervous system, which can play an important role in nerve development and neuronal differentiation by regulating microtubule dynamics and promoting axon growth and development.^33^ ADORA2A and ADORA1 are subtypes of adenosine receptors, widely distributed in various tissues and organs of the human body, highly expressed in the central system, and involved in mediating neuronal development, sleep and awakening, learning and memory, motor function and drug addiction, and a series of important processes.^34^ SLC5A1 and SLC5A2 are solute carrier family transporters, which are one of the most important membrane transporters on the human cell membrane. They play a role in the development of neuropsychiatric disorders by changing the expression, dysfunction, and regulation of neurotransmitter transporters.^35^, ^36^ AKR1B3 is a detoxification enzyme of TCM, which not only participates in plasma glucose energy metabolism and reactive oxygen species production but also in the proliferation of astrocytes through the Akt pathway to promote the repair of nerve damage.^37^ It is worth noting that there are some genes in our analysis results that have not been reported in relation to aging and the nervous system before, indicating that we can further explore the function of these genes in future experimental studies.

Given the current knowledge of these core targets, it can be inferred that gastrodin may play an antiaging role by promoting neurological development, regulating neuronal activity and function, altering the expression of neurotransmitter transporters, and participating in the proliferation of astrocytes to promote the repair of neural injury. Meanwhile, the results of GO enrichment analysis of key targets indicated that the response to substances, regulation of the sleep cycle, glucose transport and absorption, and regulation of the adenosine receptor signaling pathway play an important role in the antiaging of gastrodin. KEGG pathways enriched by these key genes mainly include galactose metabolism, metabolic pathways, and carbohydrate digestion and absorption, suggesting that gastrodin may play an antiaging role by regulating substance metabolism and carbohydrate digestion and absorption. Metabolic dysfunction may play an important role in delaying aging. Numerous studies have shown that most age‐related diseases, such as hypertension, Type 2 diabetes, cardiovascular disease, and osteoporosis, are closely associated with metabolic dysfunction.^38^, ^39^, ^40^ Metabolic interventions through calorie restriction or modulation of key metabolic signaling pathways are currently the focus of research focused on maintaining health and slowing aging.^41^, ^42^ Several studies in recent years have shown that by regulating key signaling pathways in glucose and energy metabolism, the health of various organisms can be improved and lifespan can be extended.^43^, ^44^ Some studies have reported that most of the current interventions to prevent and delay aging are achieved by targeting central metabolic pathways.^45^ In general agreement with previous studies, the results of our experiment have shown differences in the expression of aging‐related genes in the brain after gastrodin intervention. The results of network pharmacology analysis indicated that gastrodin may exert its antiaging effects by regulating signaling pathways related to substance metabolism and carbohydrate digestion and absorption. Although we have explored the targets and mechanisms of the antiaging effects of gastrodin through network pharmacology and molecular docking techniques, the underlying mechanisms are still unclear. The main limitation of this study is that no experimental validation of the screened key targets and possible mechanisms has been conducted, and it has not been determined whether gastrodin has the effect of regulating aging‐related genes. What are its antiaging targets and mechanisms of action? We will conduct further experimental studies to verify this in the follow‐up study.

## 5 CONCLUSION

In summary, in this study, aging‐related genes were detected in the hippocampus and the frontal lobe of aging mice by RT‐PCR, and the data were analyzed in combination with network pharmacology. According to the results of analyses, it is speculated that the antiaging effect of gastrodin may be related to the regulation of material metabolism and carbohydrate digestion and absorption, which provides an avenue for further verification of its mechanism in future experimental studies.

## AUTHOR CONTRIBUTIONS

Nan Zhao and Rui‐Jiang conducted animal experiments and data collection and analysis. Jun‐Jie Cheng analyzed the network pharmacology data and created pictures. Nan Zhao wrote the manuscript. Qiu‐Xia Xiao drafted and reviewed the manuscript. All authors had read and approved the final manuscript. [Correction added on 2 June 2023 after first online publication: This section was revised at the request of author.]

## CONFLICT OF INTEREST

The authors declare no conflict of interest.

## ETHICS STATEMENT

The animal experimental design in this study was approved by the Animal Experimental Ethics Review Committee of Kunming Medical University (approval no. kmmu20221547). All experimental processes complied with the guidelines for the care and use of laboratory animals published by the National Institutes of Health.

## Data Availability

The data sets used and/or analyzed during the current study are available from the corresponding author upon reasonable request.

## References

[1] Almanzar N , Antony J , Baghel AS , et al. A single‐cell transcriptomic Atlas characterizes ageing tissues in the mouse. Nature. 2020;583(7817):590‐595. 10.1038/s41586-020-2496-1 32669714 PMC8240505

[2] Kokudai Y , Honma M , Masaoka Y , et al. Cascade process mediated by left hippocampus and left superior frontal gyrus affects relationship between aging and cognitive dysfunction. BMC Neurosci. 2021;22(1):75. 10.1186/s12868-021-00680-x 34876001 PMC8650545

[3] Reagh ZM , Delarazan AI , Garber A , Ranganath C . Aging alters neural activity at event boundaries in the hippocampus and posterior medial network. Nat Commun. 2020;11(1):3980. 10.1038/s41467-020-17713-4 32769969 PMC7414222

[4] Zhang J , Xu D , Cui H , Zhao T , Chu C , Wang J . Group‐guided individual functional parcellation of the hippocampus and application to normal aging. Hum Brain Mapp. 2021;42(18):5973‐5984. 10.1002/hbm.25662 34529323 PMC8596973

[5] Singhal G , Morgan J , Jawahar MC , et al. Effects of aging on the motor, cognitive and affective behaviors, neuroimmune responses and hippocampal gene expression. Behav Brain Res. 2020;383:112501. 10.1016/j.bbr.2020.112501 31987935

[6] Huang Y , Li X , Liu Z , et al. Projections of the economic burden of care for individuals with dementia in mainland China from 2010 to 2050. PLoS One. 2022;17(2):e0263077. 10.1371/journal.pone.0263077 35113895 PMC8812891

[7] Liu E , Wang D , Sperling R , et al. Biomarker pattern of ARIA‐E participants in phase 3 randomized clinical trials with bapineuzumab. Neurology. 2018;90(10):e877‐e886. 10.1212/WNL.0000000000005060 29429971

[8] Pei H , Ma L , Cao Y , et al. Traditional Chinese medicine for Alzheimer's disease and other cognitive impairment: a review. Am J Chin Med. 2020;48(3):487‐511. 10.1142/S0192415X20500251 32329645

[9] Wang H , Yu H , Song K , Xiong F , Zhang H . Traditional Chinese Medicine for mild cognitive impairment: a protocol for systematic review and network meta‐analysis. Medicine. 2020;99(37):e22187. 10.1097/MD.0000000000022187 32925791 PMC7489630

[10] Wang ZY , Liu J , Zhu Z , et al. Traditional Chinese medicine compounds regulate autophagy for treating neurodegenerative disease: a mechanism review. Biomed Pharmacother. 2021;133:110968. 10.1016/j.biopha.2020.110968 33189067

[11] Shen CY , Jiang JG , Yang L , Wang DW , Zhu W . Anti‐ageing active ingredients from herbs and nutraceuticals used in traditional Chinese medicine: pharmacological mechanisms and implications for drug discovery. Br J Pharmacol. 2017;174(11):1395‐1425. 10.1111/bph.13631 27659301 PMC5429334

[12] Li X , Zhang Y , Wang Y , et al. The mechanisms of traditional Chinese medicine underlying the prevention and treatment of Parkinson's disease. Front Pharmacol. 2017;8:8634. 10.3389/fphar.2017.00634 PMC5609571 28970800

[13] Shen W , Fan X , Wang L , Zhang Y . Traditional Chinese medicine for post‐stroke cognitive impairment: a systematic review and meta‐analysis. Front Pharmacol. 2022;13:816333. 10.3389/fphar.2022.816333 35237166 PMC8883343

[14] Lan S , Duan J , Zeng N , et al. Network pharmacology‐based screening of the active ingredients and mechanisms of Huangqi against aging. Medicine. 2021;100(17):e25660. 10.1097/MD.0000000000025660 33907130 PMC8084007

[15] Liu Y , Gao J , Peng M , et al. A review on central nervous system effects of gastrodin. Front Pharmacol. 2018;9:24. 10.3389/fphar.2018.00024 29456504 PMC5801292

[16] Park YM , Lee BG , Park SH , et al. Prolonged oral administration of *Gastrodia elata* extract improves spatial learning and memory of scopolamine‐treated rats. Lab Anim Res. 2015;31(2):69‐77. 10.5625/lar.2015.31.2.69 26155201 PMC4490148

[17] Hsu WH , Huang NK , Shiao YJ , et al. Gastrodiae rhizoma attenuates brain aging via promoting neuritogenesis and neurodifferentiation. Phytomedicine. 2021;87:153576. 10.1016/j.phymed.2021.153576 33985879

[18] Liu CM , Tian ZK , Zhang YJ , Ming QL , Ma JQ , Ji LP . Effects of gastrodin against lead‐induced brain injury in mice associated with the Wnt/Nrf2 pathway. Nutrients. 2020;12(6):1805. 10.3390/nu12061805 32560430 PMC7353406

[19] Wang T , Chen H , Xia S , Chen X , Sun H , Xu Z . Ameliorative effect of parishin C against cerebral ischemia‐induced brain tissue injury by reducing oxidative stress and inflammatory responses in rat model. Neuropsychiatr Dis Treat. 2021;17:1811‐1823. 10.2147/NDT.S309065 34113111 PMC8187103

[20] Heese K . Gastrodia elata Blume (Tianma): hope for brain aging and dementia. Evid Based Complement Alternat Med. 2020;2020:8870148. 10.1155/2020/8870148 33424999 PMC7781687

[21] Luo K , Wang Y , Chen WS , et al. Treatment combining focused ultrasound with gastrodin alleviates memory deficit and neuropathology in an Alzheimer's disease‐like experimental mouse model. Neural Plast. 2022;2022:1‐13. 10.1155/2022/5241449 PMC8776436 35069727

[22] Shi R , Zheng C , Wang H , et al. Gastrodin alleviates vascular dementia in a 2‐VO‐Vascular dementia rat model by altering amyloid and tau levels. Pharmacology. 2020;105(7‐8):386‐396. 10.1159/000504056 31752010

[23] Liu LF , Song JX , Lu JH , et al. Tianma Gouteng Yin, a Traditional Chinese medicine decoction, exerts neuroprotective effects in animal and cellular models of Parkinson's disease. Sci Rep. 2015;5:16862. 10.1038/srep16862. PMID: 26578166; PMCID: PMC4649620. 26578166 PMC4649620

[24] Wang X , Wang ZY , Zheng JH , LI S . TCM network pharmacology: a new trend towards combining computational, experimental and clinical approaches. Chin J Nat Med. 2021;19(1):1‐11. 10.1016/S1875-5364(21)60001-8 33516447

[25] Wang YZ , Meng L , Zhuang QS , Shen L . Screening Traditional Chinese medicine combination for cotreatment of Alzheimer's disease and Type 2 diabetes mellitus by network pharmacology. J Alzheimer's Dis. 2021;80(2):787‐797. 10.3233/JAD-201336 33579846

[26] Ye X , Wang H , Cheng S , Xia L , Xu X , Li X . Network pharmacology‐based strategy to investigate the pharmacologic mechanisms of coptidis rhizoma for the treatment of Alzheimer's disease. Front Aging Neurosci. 2022;14:890046. 10.3389/fnagi.2022.890046 35795239 PMC9252849

[27] Wu HB , Xiao YG , Chen JS , Qiu ZK . The potential mechanism of Bupleurum against anxiety was predicted by network pharmacology study and molecular docking. Metab Brain Dis. 2022;37(5):1609‐1639. 10.1007/s11011-022-00970-1 35366129

[28] Chen B , Fan W , Liu J , Wu FX . Identifying protein complexes and functional modules—from static PPI networks to dynamic PPI networks. Brief Bioinform. 2014;15(2):177‐194. 10.1093/bib/bbt039 23780996

[29] XiYang YB , Wang YC , Zhao Y , et al. Sodium channel voltage‐gated beta 2 plays a vital role in brain aging associated with synaptic plasticity and expression of COX5A and FGF‐2. Mol Neurobiol. 2016;53(2):955‐967. 10.1007/s12035-014-9048-3 25575679

[30] Tan YX , Hong Y , Jiang S , et al. MicroRNA‑449a regulates the progression of brain aging by targeting SCN2B in SAMP8 mice. Int J Mol Med. 2020;45(4):1091‐1102. 10.3892/ijmm.2020.4502 32124967 PMC7053848

[31] Nykjaer A , Willnow TE . Sortilin: a receptor to regulate neuronal viability and function. Trends Neurosci. 2012;35(4):261‐270. 10.1016/j.tins.2012.01.003 22341525

[32] Xu SY , Jiang J , Pan A , Yan C , Yan XX . Sortilin: a new player in dementia and Alzheimer‐type neuropathology. Biochem Cell Biol. 2018;96(5):491‐497. 10.1139/bcb-2018-0023 29687731

[33] Yang M , Wu M , Xia P , et al. The role of microtubule‐associated protein 1B in axonal growth and neuronal migration in the central nervous system. Neural Regen Res. 2012;7(11):842‐848. 10.3969/j.issn.1673-5374.2012.11.008 25737712 PMC4342712

[34] Jaberi E , Rohani M , Shahidi GA , et al. Mutation in ADORA1 identified as likely cause of early‐onset parkinsonism and cognitive dysfunction. Mov Disorders. 2016;31(7):1004‐1011. 10.1002/mds.26627 27134041

[35] Jia Y , Wang N , Zhang Y , Xue D , Lou H , Liu X . Alteration in the function and expression of SLC and ABC transporters in the neurovascular unit in Alzheimer's disease and the clinical significance. Aging Dis. 2020;11(2):390‐404. 10.14336/AD.2019.0519 32257549 PMC7069460

[36] Ayka A , Şehirli AÖ . The role of the SLC transporters protein in the neurodegenerative disorders. Clin Psychopharmacol Neurosci. 2020;18(2):174‐187. 10.9758/cpn.2020.18.2.174 32329299 PMC7236796

[37] Chen X , Chen C , Hao J , et al. AKR1B1 upregulation contributes to neuroinflammation and astrocytes proliferation by regulating the energy metabolism in rat spinal cord injury. Neurochem Res. 2018;43(8):1491‐1499. 10.1007/s11064-018-2570-3 29948725

[38] Oliveira V , Kwitek AE , Sigmund CD , Morselli LL , Grobe JL . Recent advances in hypertension: intersection of metabolic and blood pressure regulatory circuits in the central nervous system. Hypertension. 2021;77(4):1061‐1068. 10.1161/HYPERTENSIONAHA.120.14513 33611936 PMC7990288

[39] Derosa G , Catena G , Gaudio G , D'Angelo A , Maffioli P . Adipose tissue dysfunction and metabolic disorders: is it possible to predict who will develop type 2 diabetes mellitus? Role of markers in the progression of dIabeteS in obese patients (the RESISTIN trial). Cytokine. 2020;127:154947. 10.1016/j.cyto.2019.154947 31811995

[40] Guo Y , Yang J , Ma R , et al. Metabolic dysfunction‐associated fatty liver disease is associated with the risk of incident cardiovascular disease: a prospective cohort study in Xinjiang. Nutrients. 2022;14(12):2361. 10.3390/nu14122361 35745091 PMC9231197

[41] Smith HJ , Sharma A , Mair WB . Metabolic communication and healthy aging: where should we focus our energy? Dev Cell. 2020;54(2):196‐211. 10.1016/j.devcel.2020.06.011 32619405 PMC8168458

[42] Liang Y , Gao Y , Hua R , et al. Calorie intake rather than food quantity consumed is the key factor for the anti‐aging effect of calorie restriction. Aging. 2021;13(17):21526‐21546. 10.18632/aging.203493 34493691 PMC8457579

[43] Parkhitko AA , Filine E , Mohr SE , Moskalev A , Perrimon N . Targeting metabolic pathways for extension of lifespan and healthspan across multiple species. Ageing Res Rev. 2020;64:101188. 10.1016/j.arr.2020.101188 33031925 PMC9038119

[44] Smith WK , Ingram DK , de Cabo R , Pasquina P . Metabolic pathways and therapeutics to promote resilience, rehabilitation and delayed aging. GeroScience. 2021;43(3):1069‐1070. 10.1007/s11357-021-00371-9 33909238 PMC8190427

[45] Angoff R , Himali JJ , Maillard P , et al. Relations of metabolic health and obesity to brain aging in young to middle‐aged adults. J Am Heart Assoc. 2022;11(6):e022107. 10.1161/JAHA.121.022107 35229662 PMC9075324

